# Combined Anterior Cruciate Ligament and Anterolateral Ligament Reconstruction Using a Flexible Reaming System

**DOI:** 10.1016/j.eats.2024.103213

**Published:** 2024-08-24

**Authors:** Alejandro Jaramillo Quiceno, Paula Andrea Sarmiento Riveros, Camilo Partezani Helito, Andre Giardino Moreira da Silva, Rubén Darío Arias Pérez, Ricardo Londoño García

**Affiliations:** a Orthopedic and Traumatology Service, North Clinic Foundation, Bello-Antioquia, Colombia; b Instituto de Ortopedia e Traumatologia do Hospital das Clínicas da Faculdade de Medicina da Universidade de São Paulo, São Paulo, Brazil; c Pontifical Bolivarian University, Medellín, Colombia

## Abstract

The combination of anterior cruciate ligament (ACL) and anterolateral ligament (ALL) reconstruction represents a therapeutic modality that exhibits superior clinical efficacy for certain risk groups when compared with isolated ACL reconstruction. This approach is progressively gaining broader applicability owing to its inherent prospective advantages. Despite the absence of a universally acknowledged gold-standard surgical technique, several methods have been delineated for its implementation. Typically, conventional practice involves the creation of distinct individual tunnels in the femur, followed by graft fixation using interference screws. However, the conventional steps in the technique are not without potential drawbacks. These include tunnel convergence, screw migration, screw irritation, and the risk of lateral collateral ligament injury. The manifestation of such unfavorable outcomes can necessitate a subsequent surgical intervention for effective management. Consequently, adopting a single socket-shaped tunnel strategy for concurrent reconstruction of the ACL and ALL, coupled with femoral fixation using an adjustable-loop button facilitated by a flexible reaming system, presents potential advantages. This alternative approach can mitigate the aforementioned risks by minimizing morbidity and preserving bone stock. The technique is feasible and reproducible, offering a pragmatic avenue for optimizing clinical outcomes in combined ACL and ALL reconstruction.

In the past decade, numerous studies have highlighted the advantages of incorporating an extra-articular procedure with anterior cruciate ligament (ACL) reconstruction for specific risk groups.^1^ Various surgical techniques have been outlined for combined ACL and anterolateral ligament (ALL) reconstruction, using different fixation methods and graft options. However, none has shown clear superiority.^2^

Despite the acceptance of extra-articular procedures and their potential benefits for certain at-risk patients, the indications for these interventions continue to expand. Although advantages of this approach have been reported, it is not without complications, as shown by reported reoperation rates of up to 13% after combined ACL and ALL reconstruction.^3^ Additionally, an aspect often underreported in the literature is the potential risk of injuring the femoral insertion of the lateral collateral ligament (LCL). This concern arises because the femoral insertion of the ALL is located approximately 4.7 mm proximal and posterior to the insertion point of the LCL.^4^ The risk of LCL injury can be as high as 56% when creating 9-mm tunnels at the ALL footprint on the lateral femoral condyle.^5^

Most techniques documented in the literature commonly use interference screws, buttons, or anchors for femoral fixation, but these methods are associated with potential complications such as screw migration, soft-tissue impingement, and tunnel widening.^3^^,^^6^ Additionally, the conventional practice of establishing individual tunnels for each ligament may amplify morbidity and increase the risk of tunnel convergence.^5^ To address some of these risks, a flexible drill system offers a promising solution. This system allows surgeons to select the exit point of the tunnel on the external cortex of the lateral femoral condyle, drilling from inside-out and performing a single socket-shaped tunnel strategy for concurrent reconstruction of the ACL and ALL. In addition, a graft is used for both reconstructions and an adjustable-loop button fixation method is used in the lateral femoral cortex, so this approach can mitigate the aforementioned risks, minimizing morbidity and preserving bone stock.

The outcomes of combined reconstruction of the ACL and ALL have shown notable success in reinstating long-term stability, decreasing the ACL failure rate, safeguarding meniscal repairs, and facilitating elevated rates of return to preinjury athletic performance.^2^ Consequently, the imperative to minimize potential complications linked with this procedure is underscored. This article aims to describe a technique for combined ACL and ALL reconstruction using a flexible reaming system to create a combined femoral tunnel, with fixation achieved through an adjustable-loop cortical button on the femur.

## Surgical Technique

### Patient Positioning and Setup

**Fig 1:**
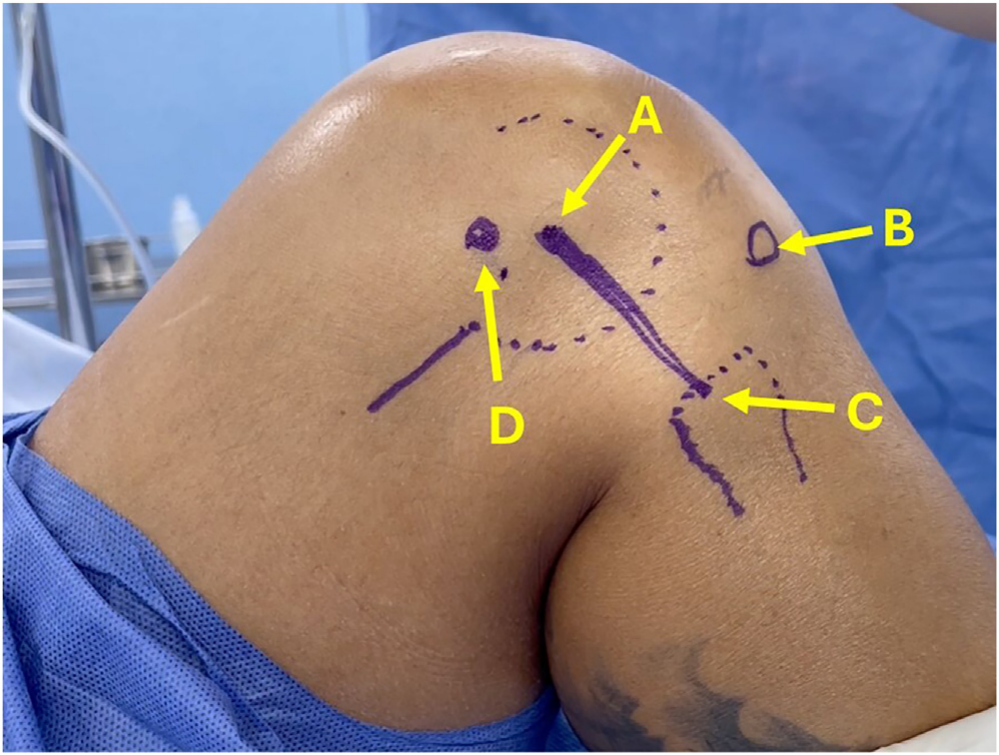
Lateral view of right knee. (A) Fibular head. (B) Gerdy tubercle. (C) Lateral femoral epicondyle. (D) Insertion point of anterolateral femoral ligament.

The patient is placed in the supine position, and a tourniquet is appropriately applied to the lower extremity. The precise markings of the fibular head, Gerdy tubercle, and lateral epicondyle are meticulously drawn on the patient’s skin (Fig 1).

### Graft Harvesting and Preparation

**Fig 2:**
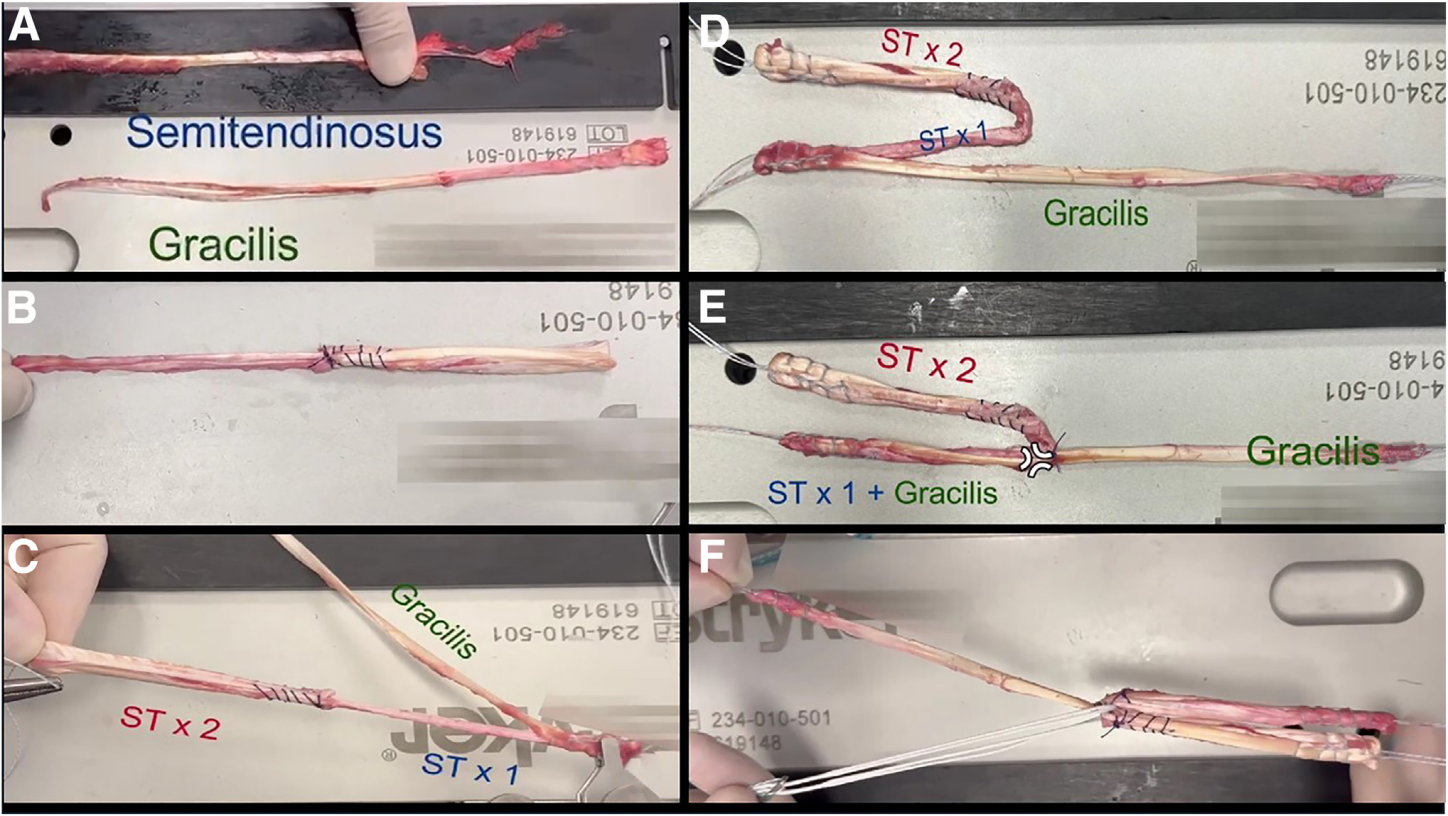
Graft preparation steps. (A) Graft preparation is performed using semitendinosus and gracilis autograft. (B) Initially, the total length of the semitendinosus is measured. Then, it is divided into 3 equal parts, and 2 parts of this graft are united using an absorbable suture. (C-E) One end of the semitendinosus (ST) graft is attached to the gracilis, and the free strands are sutured. Krackow stitches are used for graft preparation. (F) The adjustable-loop fixation device is placed on the anterior cruciate ligament portion of the graft.

Harvesting of hamstring grafts is performed as usual. A combined graft is prepared with a 3-strand semitendinosus tendon and a single-strand gracilis tendon. The remaining segment of the gracilis tendon is designated for the ALL. An adjustable-loop fixation device (ProCinch; Stryker, Kalamazoo, MI) is placed on the femoral portion of the ACL graft (Fig 2).

### Combined Femoral Tunnel Creation

**Fig 3:**
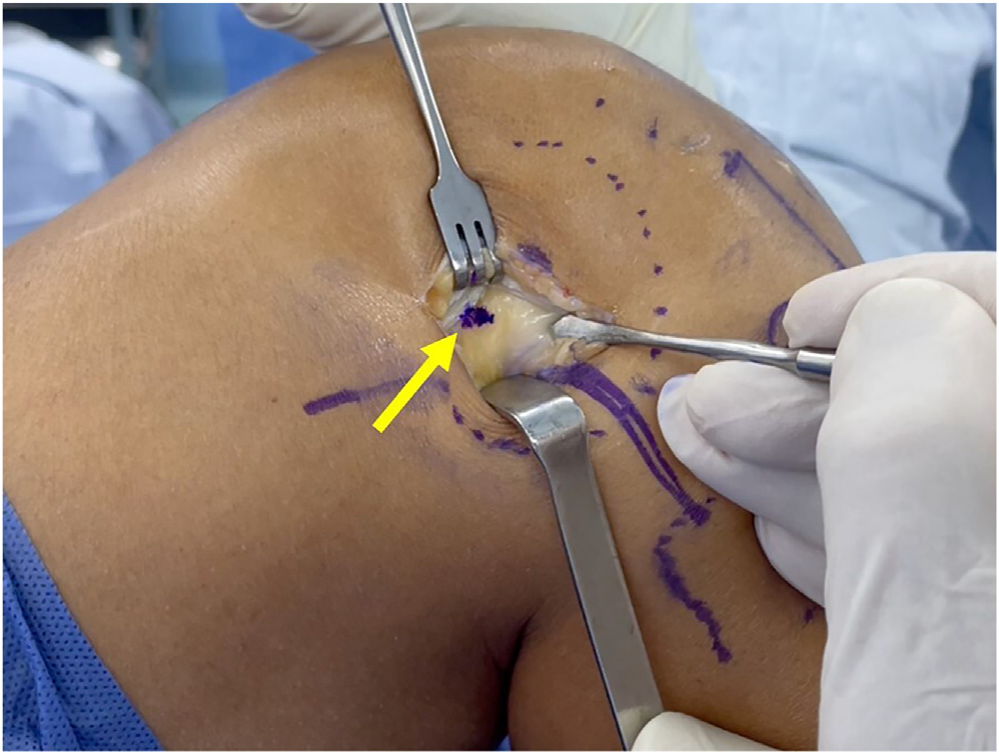
Lateral view of right knee. The yellow arrow indicates the insertion point of the anterolateral femoral ligament, which is marked after opening the iliotibial band.

A small incision is made posterior and proximal to the lateral epicondyle of the femur. Subcutaneous dissection is performed, the iliotibial band is opened, and the insertion point of the ALL is marked on the outer wall of the lateral femoral condyle (Fig 3).

Conventional arthroscopic anterolateral and anteromedial portals are created, complemented by a central transpatellar portal. The camera is inserted through the lateral portal while hand instruments are introduced through the medial portal, facilitating arthroscopic exploration. Thorough joint cleaning enhances visualization, and a comprehensive visual inspection is conducted to rule out any associated lesions.

**Fig 4:**
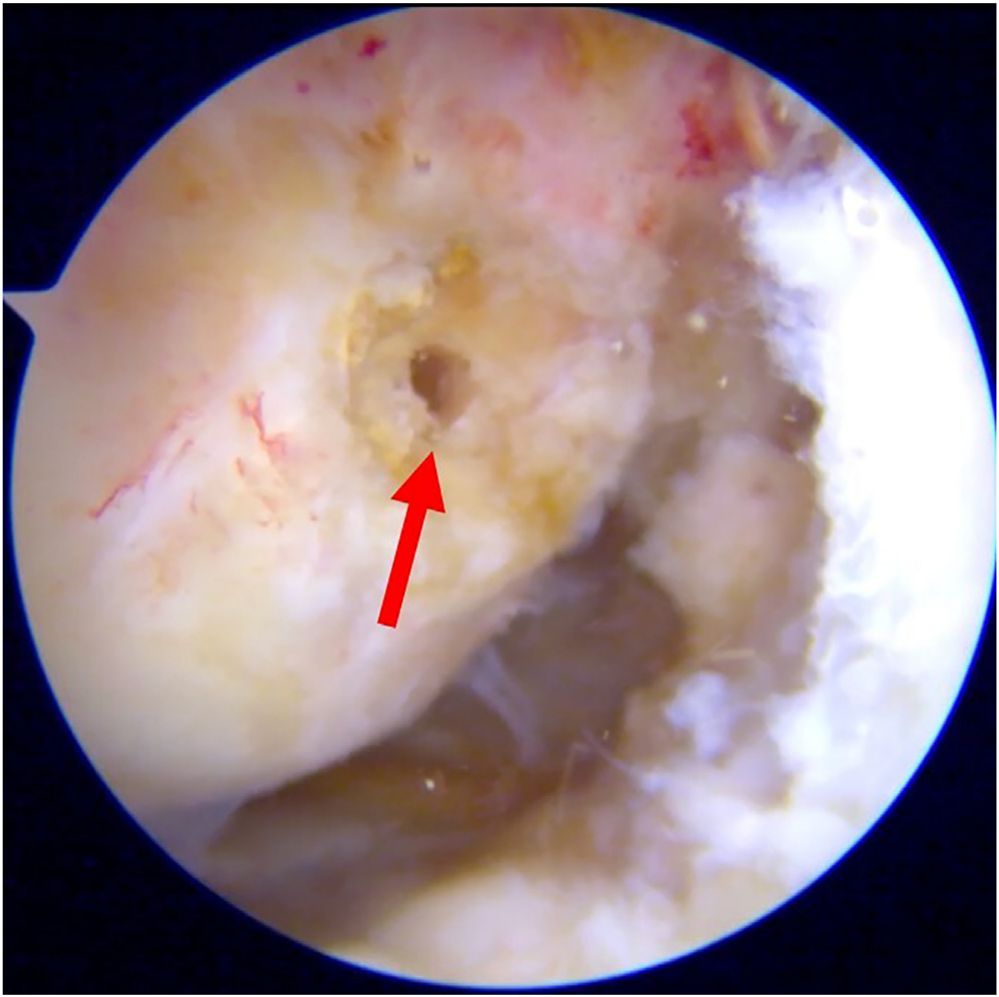
Arthroscopic view of right knee. The red arrow indicates the femoral insertion point of the anterior cruciate ligament.

**Fig 5:**
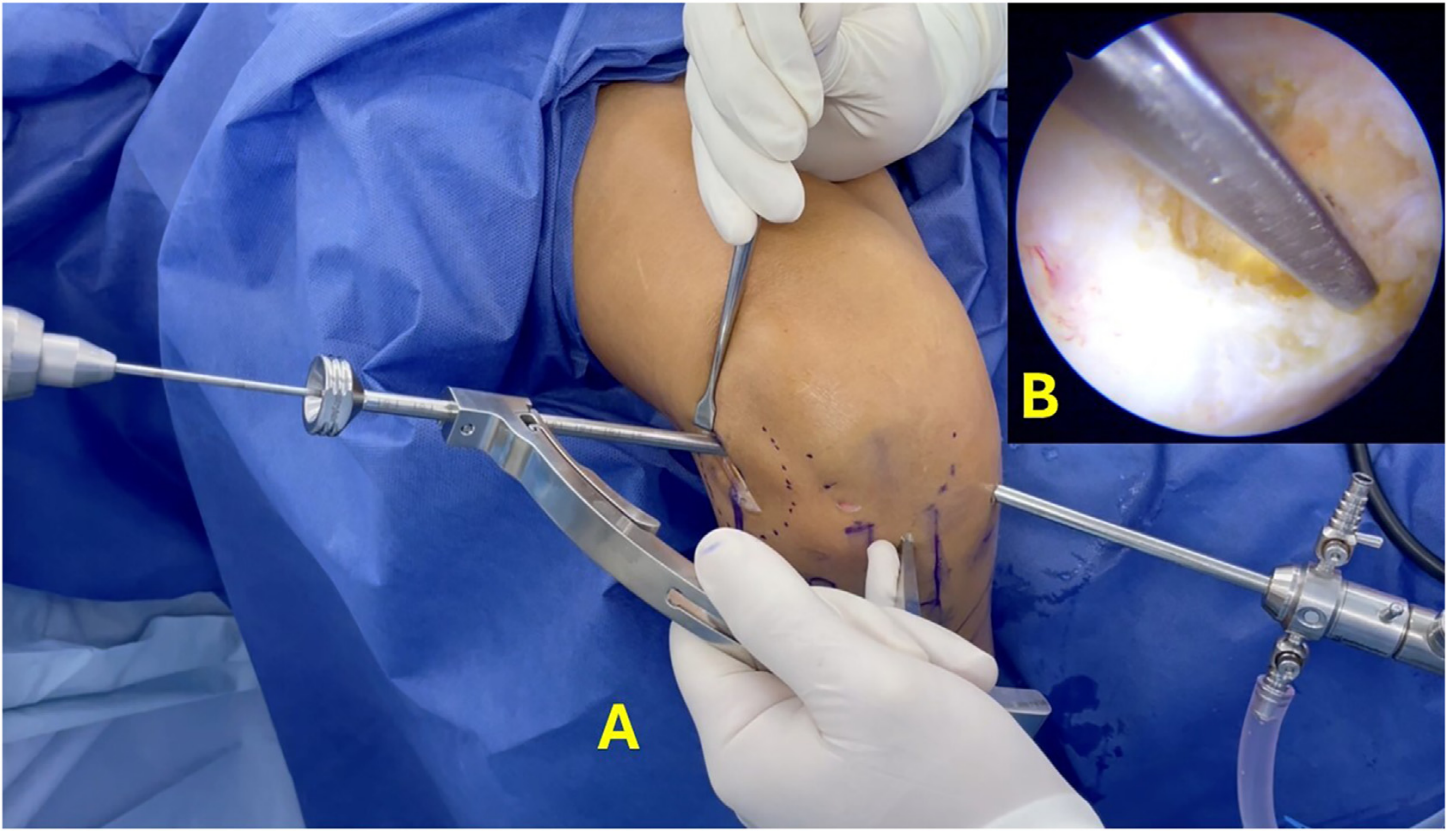
(A) Anterolateral view of right knee. The camera is inserted through the medial portal. The guide is positioned from the transpatellar portal at the previously marked points, internally at the femoral insertion of the anterior cruciate ligament and externally at the femoral insertion of the anterolateral ligament. (B) Arthroscopic view of internal position of guide.

Subsequently, the lateral wall of the intercondylar notch is prepared, and the anatomic location of the ACL is identified. The Stryker VersiTomic Flexible ACL Reaming System is used for this technique. The curved guide of the flexible reaming system is positioned on the femoral footprint of the ACL, and a flexible guidewire is inserted 0.5 cm to mark this point (Fig 4). Then, the conventional outside-in guide is positioned internally on the ACL footprint and externally on the previously marked point of the ALL on the lateral femoral condyle, and a guidewire is passed from outside-in (Fig 5). The position of the guidewire is checked, and the width of the lateral femoral condyle is measured. Then, a femoral tunnel of 5 mm in diameter is drilled from outside-in (Video 1).

### ALL Tibial Tunnel Creation

**Fig 6:**
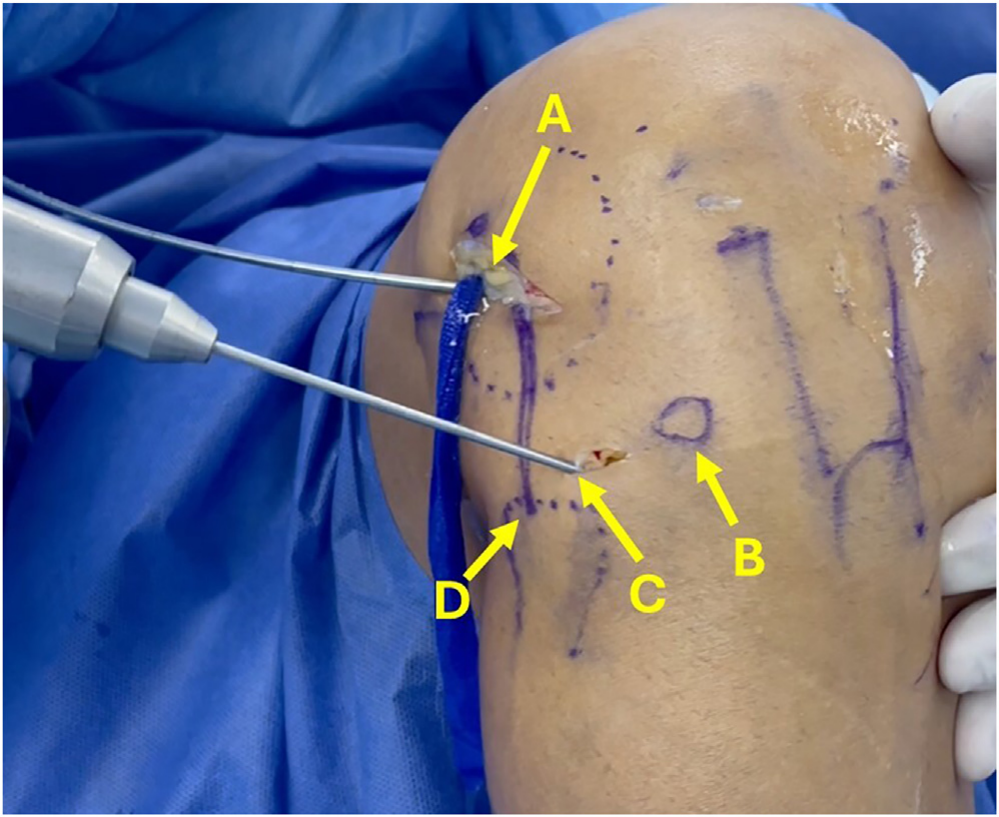
Anterolateral view of right knee. A guidewire is passed between the midpoint of the Gerdy tubercle and the head of the fibula toward the anteromedial surface of the tibia. (A) Guidewire located at femoral insertion of anterolateral ligament. (B) Gerdy tubercle. (C) Guidewire located at tibial insertion of anterolateral ligament. (D) Head of fibula.

**Fig 7:**
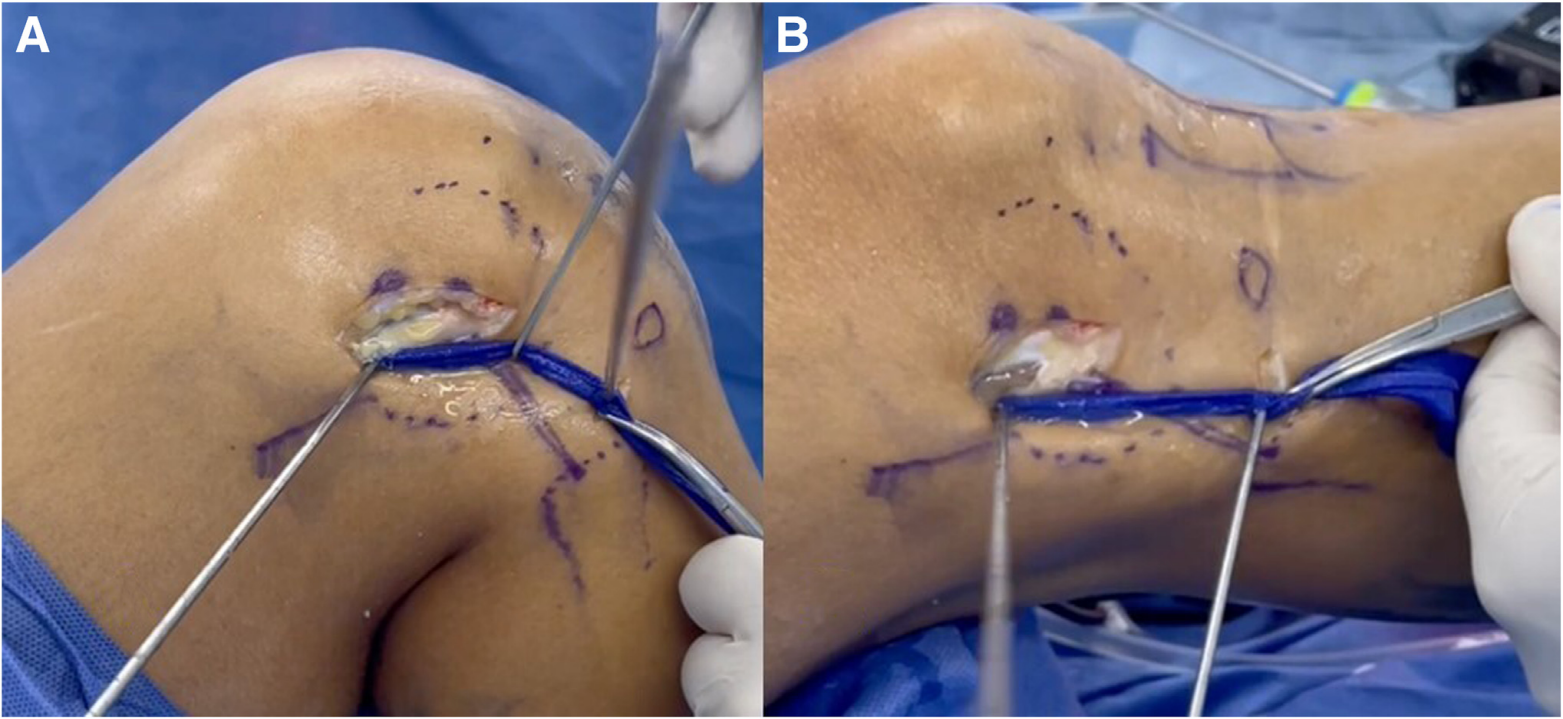
Lateral view of right knee. With the guidewires placed at the anterolateral ligament insertions, tape is used to simulate the function of the ligament. (A) Loose in flexion. (B) Tight in extension.

A small incision is made 1 cm distal to the joint line and at the midpoint between the Gerdy tubercle and the head of the fibula. A guidewire is positioned at the ALL tibial footprint and passed toward the anteromedial surface of the tibia (Fig 6). The position of the ALL tunnels is checked. A tape is used to verify proper functioning of the ALL, tight in extension and loose in flexion (Fig 7). If the position is appropriate, the ALL tibial tunnel is drilled over the guidewire, and a suture is looped through the tunnel.

### ACL Femoral Socket Creation

**Fig 8:**
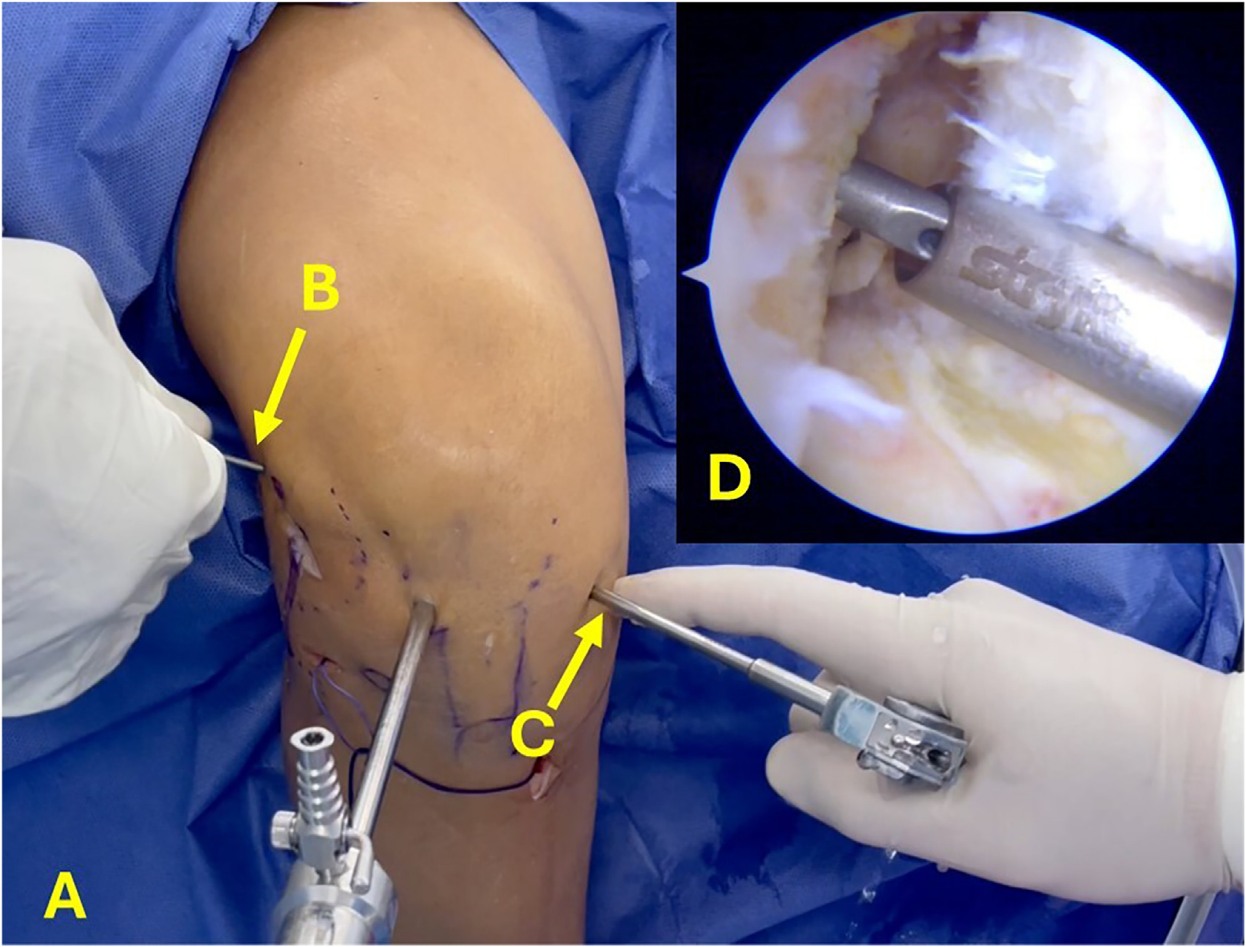
(A-C) Anterolateral view of right knee. The camera is inserted from the lateral portal (A), the flexible guidewire is inserted from outside-in through the previously drilled femoral tunnel (B), and the flexible guidewire is retrieved through the medial portal (C). (D) Arthroscopic view showing intra-articular retrieval of flexible guidewire inserted from outside.

**Fig 9:**
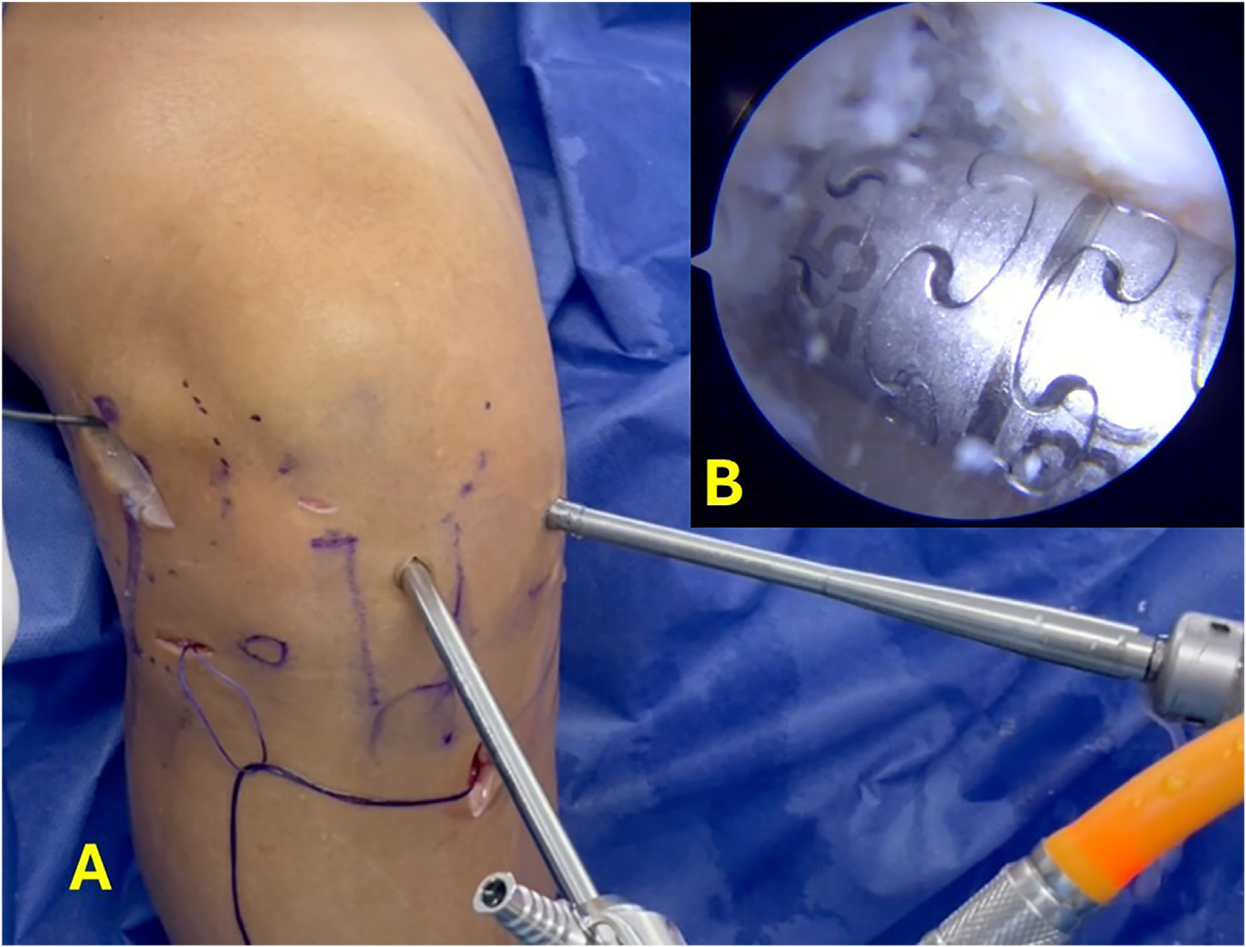
(A) Anterolateral view of right knee. The camera is inserted from the transpatellar portal. By use of the flexible reamer system, the femoral socket is drilled from inside-out to a length of 25 mm. (B) Arthroscopic view of femoral drilling.

To create the ACL femoral socket, the curved guidewire of the flexible system is inserted into the previously drilled 5-mm femoral tunnel from outside-in. The curved femoral guide is positioned on the inner exit of the tunnel to guide the flexible guidewire through the anteromedial portal (Fig 8). The ACL femoral socket is drilled from inside-out using the flexible reamer system, with the measured diameter of the ACL portion of the graft and a length of 25 mm, preserving the external cortex of the lateral femoral condyle (Fig 9). Then, a loop suture is passed through the tunnel to guide passage of the graft and fixation device.

### ACL Tibial Tunnel Creation

The tibial tunnel for the ACL is made conventionally, aiming at the footprint of the native ACL (Video 1).

### Graft Passage and Fixation

**Fig 10:**
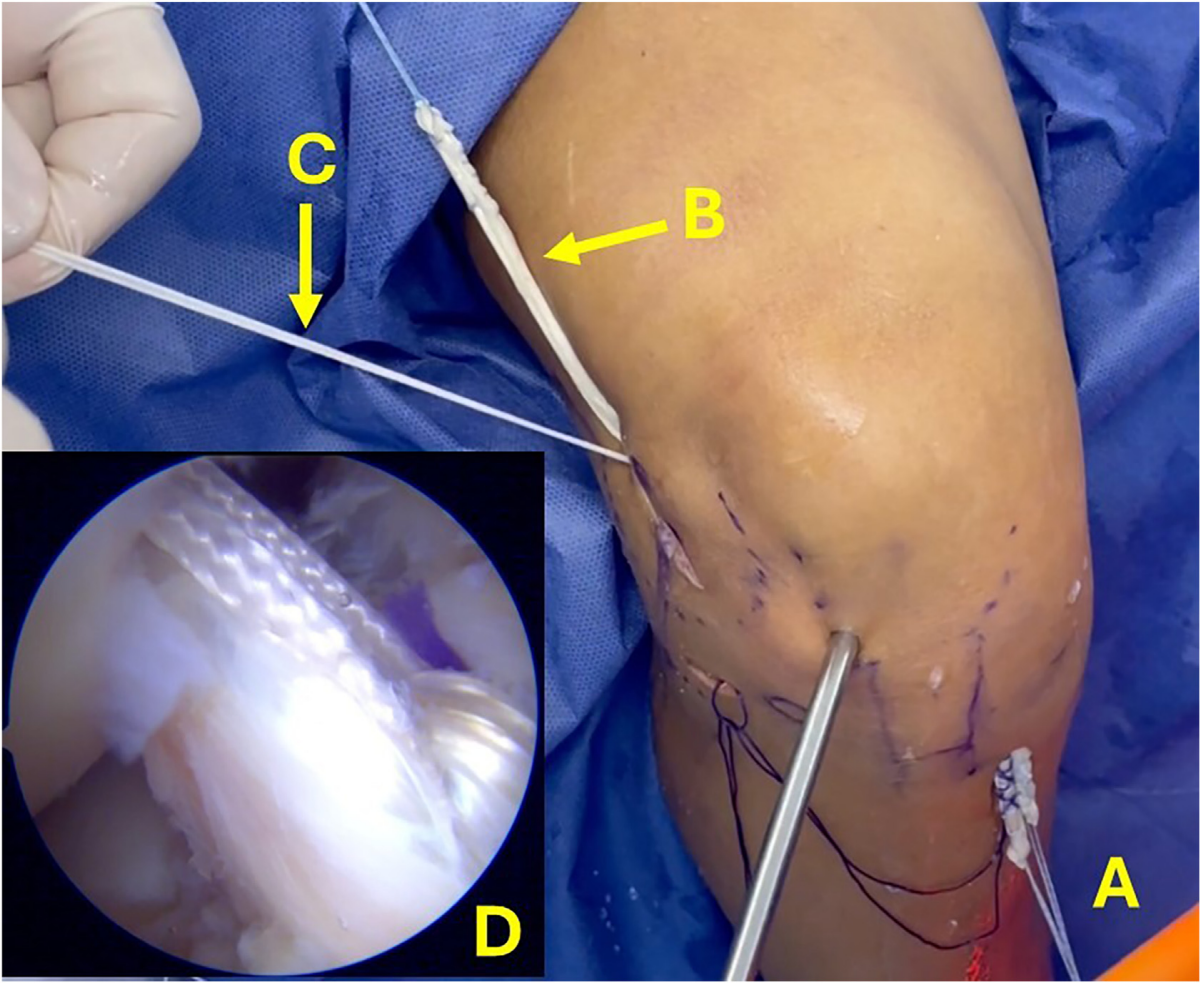
(A-C) Anterolateral view of right knee, with camera inserted through lateral portal. (A) Insertion of graft through anterior cruciate ligament tunnels. (B) Gracilis graft used to reconstruct lateral anterolateral ligament. (C) Threads of adjustable-loop button. (D) Arthroscopic view showing passage of graft within joint.

**Fig 11:**
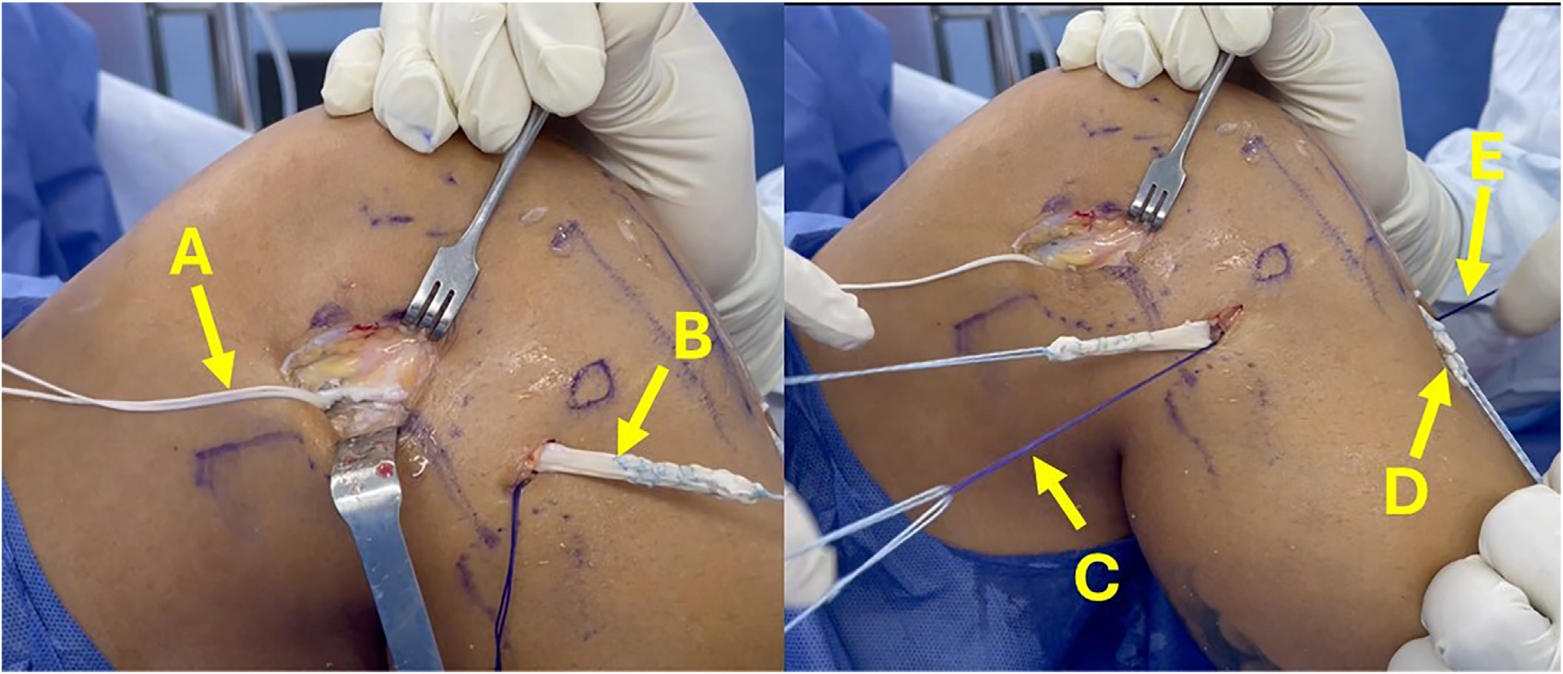
Lateral view of right knee. (A) Threads of adjustable-loop anterior cruciate ligament button. (B) Gracilis graft under iliotibial band passed from femur to tibia. (C) Passage of initial portion of suture loop through tibial tunnel for anterolateral ligament reconstruction. (D) Anterior cruciate ligament graft in tibial tunnel. (E) Final portion of suture loop through tibial tunnel for anterolateral ligament.

The graft is threaded through the tunnels using loop sutures. Once the button is flipped and securely fastened to the lateral cortex of the femur, the tensioning sutures are gradually tightened to advance the graft into the femoral socket (Fig 10). Subsequently, a loop suture is passed under the iliotibial band to guide the passage of the ALL graft from the femur toward the tibial incision. Then, the ALL graft is inserted into the ALL tibial tunnel, exiting on the anteromedial surface of the tibia (Fig 11).

**Fig 12:**
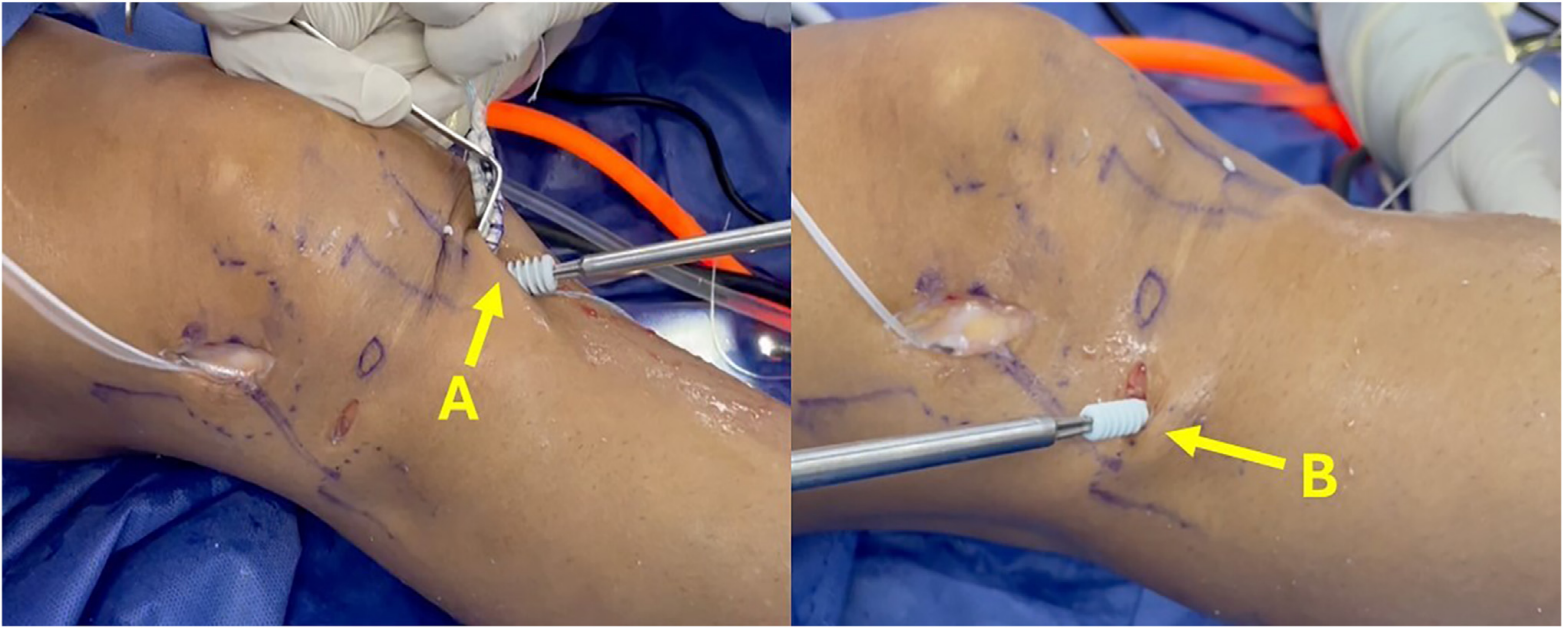
Anterior view of right knee showing tibial fixation of ligaments. (A) Interference screw fixation of anterior cruciate ligament at 20° of flexion after pre-tensioning of graft. (B) Tibial fixation of gracilis graft with interference screw in full extension.

After pre-tensioning of the ACL graft, tibial fixation of the ACL is performed with an interference screw, at 20° of flexion, accompanied by the application of a posterior drawer force to the tibia. Finally, tibial fixation of the ALL is carried out using an interference screw (Biosteon; Stryker), with the knee in full extension and neutral rotation (Fig 12).

## Discussion

The proposed technique stands out for its capability to create a single femoral socket-shaped tunnel for simultaneous reconstruction of the ACL and ALL. This is achieved through an inside-out approach using a flexible reamer system (VersiTomic), which offers the advantage of preserving bone stock for potential revision surgery. Additionally, it reduces the need for excessive knee hyperflexion during drilling. Grafts are secured to the femur using an adjustable-loop button, potentially reducing the risks associated with interference screws.^6^ Furthermore, this approach lowers the risk of LCL injury and theoretically decreases the chance of harming the lateral inferior genicular arteries and peroneal nerve by avoiding the use of an iliotibial band graft.^5^^,^^7^ The surgical procedure involves minor incisions, improving esthetic outcomes, and minimizing postoperative discomfort (Table 1). This method not only enhances precision and flexibility in tunnel creation but also allows for the establishment of an anatomically accurate tunnel position.

**Table 1:** Advantages and Disadvantages

Advantages
Anterior cruciate ligament and anterolateral ligament reconstruction is performed with a combined femoral tunnel using 1 graft.
The technique allows optimal anatomic adaptability.
Instead of the creation of full tunnels, a single socket-shaped tunnel is created in the femur.
The femoral cortex tunnel exhibits a reduced diameter, safeguarding the femoral insertion of the lateral collateral ligament.
There is a diminished potential for irritation caused by femoral hardware.
Bone stock is preserved for potential revision surgery.
The technique involves minor incisions, enhancing the esthetic result.
The flexible reaming system has a lower cost than a retrograde drill.
Disadvantages
Specialized instruments are needed, specifically the flexible reaming system.
The technique necessitates the harvesting of hamstring autografts.
It is possible to have a small graft diameter.

To achieve combined ACL and ALL reconstruction with a single femoral socket-shaped tunnel and fixation using a cortical button, a technique allowing precise tunnel positioning is essential. The conventional anteromedial portal technique with rigid instruments fails to create the combined tunnel because it cannot accurately predict the exit point of the femoral tunnel in the lateral cortex.^8^ Quiceno et al.^7^ described a method for combined ACL and ALL reconstruction with a single femoral socket-shaped tunnel using retrograde drill devices. This article proposes an alternative technique using a flexible reaming system, which offers the advantage of lower cost compared with the retrograde drill (Table 1).

Possible complications of our procedure mirror those of ACL and ALL reconstruction, encompassing the risks of rerupture, arthrofibrosis, infection, loss of extension, and potential non-isometric tunnel placement leading to knee stiffness. It is crucial to preoperatively identify and plan appropriate bony landmarks in all cases. Additionally, using a strand for ALL reconstruction may slightly reduce the diameter of the ACL graft, which must be maintained at a minimum of 8 mm^2^ (Table 2).

**Table 2:** Pearls and Pitfalls

Pearls
It is crucial to preoperatively identify and plan appropriate bony landmarks in all cases.
Using high-strength sutures when preparing the anterolateral ligament end of the gracilis graft can help prevent graft rupture when passing the interference screw through the thick cortex of the tibia.
After graft fixation, the remaining sutures at the ends of both the anterior cruciate ligament and anterolateral ligament grafts can be securely tied, offering secondary fixation for both grafts.
Pitfalls
During the creation of the femoral socket, surgeons must be cautious of the proximity of the guidewire to the medial femoral condyle. It is essential to take care to prevent iatrogenic damage to the articular cartilage when passing reamers from the anteromedial portal.
During positioning of the guide for the combined femoral tunnel, it is important to exercise caution to avoid excessively posterior placement of the guide. This helps prevent violation of the posterior wall of the condyle.

The implementation of the described technique for simultaneous reconstruction of the ACL and ALL holds the potential to signify an advancement in enhancing clinical outcomes and improving the overall patient experience when compared with traditional methods. The presented technique is both viable and reproducible, making it an excellent option for surgeons performing ACL reconstruction with indications for an associated extra-articular procedure. However, future clinical studies are necessary to establish the long-term outcomes of this technique.

## Disclosures

The authors declare the following financial interests/personal relationships which may be considered as potential competing interests: A.J.Q. receives speaking and lecture fees from 10.13039/100004331Johnson & Johnson Services and Stryker. P.A.S.R. receives speaking and lecture fees from Arthrex. C.P.H. receives speaking and lecture fees from 10.13039/100009026Smith & Nephew, ConMed, and Johnson & Johnson Services. All other authors (A.G.M.d.S., R.D.A.P., R.L.G.) declare that they have no known competing financial interests or personal relationships that could have appeared to influence the work reported in this paper.

## Supplementary Data

Video 1 Surgical technique. This technique allows the execution of a single femoral socket-shaped tunnel for reconstruction of both the anterior cruciate ligament (ACL) and anterolateral ligament (ALL) through an inside-out approach using a flexible reamer system. Additionally, graft fixation to the femur is achieved using an adjustable-loop button.
